# Synergistic Effect of Co_3_(HPO_4_)_2_(OH)_2_ Cocatalyst and Al_2_O_3_ Passivation Layer on BiVO_4_ Photoanode for Enhanced Photoelectrochemical Water Oxidation

**DOI:** 10.3390/molecules29030683

**Published:** 2024-02-01

**Authors:** Zijun Sun, Zhen Li, Jinlin Chen, Yuying Yang, Chunrong Su, Yumin Lv, Zhenhong Lu, Xiong He, Yongqing Wang

**Affiliations:** Guangxi Key Laboratory of Multidimensional Information Fusion for Intelligent Vehicles, School of Electronic Engineering, Guangxi University of Science and Technology, Liuzhou 545000, China; sunzijun@gxust.edu.cn (Z.S.); bzsbcqlz@126.com (Z.L.); 15777785956@163.com (C.S.);

**Keywords:** photoelectrochemical, Co_3_(HPO_4_)_2_(OH)_2_, oxygen evolution catalyst, BiVO_4_, photoanode

## Abstract

Bismuth vanadate (BVO) is regarded as an exceptional photoanode material for photoelectrochemical (PEC) water splitting, but it is restricted by the severe photocorrosion and slow water oxidation kinetics. Herein, a synergistic strategy combined with a Co_3_(HPO_4_)_2_(OH)_2_ (CoPH) cocatalyst and an Al_2_O_3_ (ALO) passivation layer was proposed for enhanced PEC performance. The CoPH/ALO/BVO photoanode exhibits an impressive photocurrent density of 4.9 mA cm^−2^ at 1.23 V_RHE_ and an applied bias photon-to-current efficiency (ABPE) of 1.47% at 0.76 V_RHE_. This outstanding PEC performance can be ascribed to the suppressed surface charge recombination, facilitated interfacial charge transfer, and accelerated water oxidation kinetics with the introduction of the CoPH cocatalyst and ALO passivation layer. This work provides a novel and synergistic approach to design an efficient and stable photoanode for PEC applications by combining an oxygen evolution cocatalyst and a passivation layer.

## 1. Introduction

Photoelectrochemical (PEC) water splitting is recognized as an attractive approach for converting inexhaustible solar energy into hydrogen energy [1,2,3,4]. In a typical PEC system, the oxygen evolution reaction (OER) occurs at the photoanode, and the hydrogen evolution reaction (HER) occurs at the counter electrode. Owing to the characteristics of the material itself, there is currently no single semiconductor material that can satisfy these two half-reactions at the same time, so the anode and cathode in the PEC system often use different semiconductor materials. At the core of the PEC water splitting is the OER reaction, a key challenge in PEC water splitting which involves a complex four-proton coupled multi-electron process. Moreover, the high performance of a photoanode is predominantly determined by its ability to absorb solar light, facilitate charge separation, and exhibit high catalytic activity and stability during the PEC water splitting. Among the variety of photoanode materials, BiVO_4_ (BVO) is recognized as one of the most promising choices for PEC water splitting due to its unique qualities, such as low cost, coupled with a narrow band gap (~2.4 eV) that renders it an optimal absorber of visible light, essential for the efficient harnessing of solar energy. Moreover, BVO’s advantageous positioning of its appropriate valence band greatly aids in the water oxidation process, a key step in PEC. These characteristics elevate BVO as a promising candidate for sustainable hydrogen production [5,6]. However, the rapid charge recombination, slow surface water oxidation, and severe photocorrosion limit its PEC water splitting performance [7,8,9]. To overcome these limitations, a variety of strategies have been employed to enhance the PEC performance of BVO-based photoanodes, such as morphology control [10], element doping [11], heterojunctions formation [12,13], and oxygen evolution catalysts (OECs) modification [14,15].

A promising strategy to facilitate charge separation and enhance the oxidation reaction kinetics involves the incorporation of suitable OECs because it could facilitate the charge separation, accelerate the water oxidation kinetics, and restrain photocorrison [16]. To date, numerous transition metal-based materials have been considerably utilized as OECs due to their excellent catalytic properties and earth abundance [17]. For example, Khiarak et al. introduced electrochemically reduced graphene oxide (ERGO)/sulfur-doped copper oxide supported with carbon cloth (CC), serving as an extremely efficient OER electrocatalyst [18]. Among the multitude of reported OECs, cobalt-based materials have been widely explored owing to their excellent water oxidation activity [17]. Within these cobalt-based OECs, cobalt hydroxides have attracted significant interest due to their favorable combination of moderate electronic conductivity and high catalytic activity [19]. For instance, Ning et al. deposited the Co(OH)_2_ as a cocatalyst on the BVO photoanode to obtain a higher photocurrent density. This improvement is attributed to the incorporation of Co(OH)_2_ to rapidly migrate photogenerated holes to its surface and effectively inhibit interface recombination [20]. As one of the most important electrocatalytic oxygen evolution catalysts, CoPi is often employed to modify semiconductor photoanodes due to its superior activity, low overpotential and inherent self-repair properties [21]. Hernandez et al. introduced CoPi on the BVO photoanode, achieving a high photocurrent density of 3.0 mA cm^−2^ at 1.23 V_RHE_. This improvement is attributed to CoPi’s role as an effective oxygen evolution catalyst [22]. Reddy et al. used CoPi to modify the BVO photoanode to achieve a notable photocurrent density of 2.7 mA cm^−2^ and a negative shift in the onset potential to 0.32 V_RHE_, enhancing separation and suppressing recombination of charge carriers [23]. The phosphate group on the catalyst acts as a proton acceptor, which contributes to the self-repair of the catalyst and assists the proton–electron transfer process during metal oxidation, thereby improving the water oxidation activity [24]. However, the dissolution of CoPi under specific pH conditions significantly hinders its long-term stability, impacting its efficiency in PEC systems [25].

Recently, we reported that nickel nitrate hydroxides exhibit superior performance in electrocatalytic water oxidation processes compared to nickel hydroxides and nickel salts. According to our previous work, we found that transition metal basic salts have better electronic conductivity and more active sites, leading to the rapid charge transfer and fast water oxidation. Moreover, the presence of hydroxide groups could effectively enhance its chemical stability [26]. Consequently, even though the incorporation of transition metal basic salts in photoanode modification for PEC water splitting remains largely unexplored, their prospective role as competent and stable OECs is increasingly recognized. These materials, with their unique characteristics, are unitized to significantly enhance the PEC performance of photoanodes. Accordingly, they provide important advantages, including enhanced charge transfer capabilities and heightened durability under stringent operational conditions, which are critical for the PEC progression and technologies. This emerging recognition underscores the potential of transition metal basic salts to revolutionize solar fuel production. In addition, to suppress the photocorrosion and modify the surface state distribution of the BVO photoanode, a passivation layer was fabricated between the OECs and semiconductor [27]. The passivation layer is usually composed of an ultrathin layer of metal oxide, such as Al_2_O_3_ (ALO) [28], ZnO [29], NiO_x_ [30], and so on. Among them, Al_2_O_3_ shows the best passivation effect and low cost [31].

**Figure 1:**
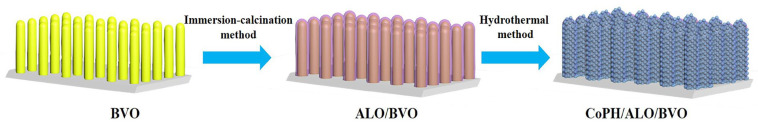
Schematic of the synthetic procedure for the CoPH/ALO/BVO photoanode.

Herein, we first report that the ALO passivation layer and Co_3_(HPO_4_)_2_(OH)_2_ (CoPH) OEC were synthesized on BVO (synthesized based on previous report [14]) surfaces by a convenient immersion–calcination method and a hydrothermal method, respectively (Figure 1). CoPH demonstrated superior enhanced properties in comparison to CoPi and Co(OH)_2_. Further PEC analysis indicated that the CoPH/ALO/BVO photoanode exhibits an impressive photocurrent density, reaching 4.9 mA cm^−2^ at 1.23 V_RHE_. This performance makes a significant advancement in the field of PEC water splitting. In addition, we observed that the stability of the CoPH/ALO/BVO photoanode was substantially superior to that of the bare BVO photoanode. This improvement in stability is critical for practical applications. These findings provide new avenues for the development of highly efficient photoanodes for solar energy conversion, providing the way toward more sustainable and eco-friendly energy solutions.

## 2. Results and Discussion

**Figure 2:**
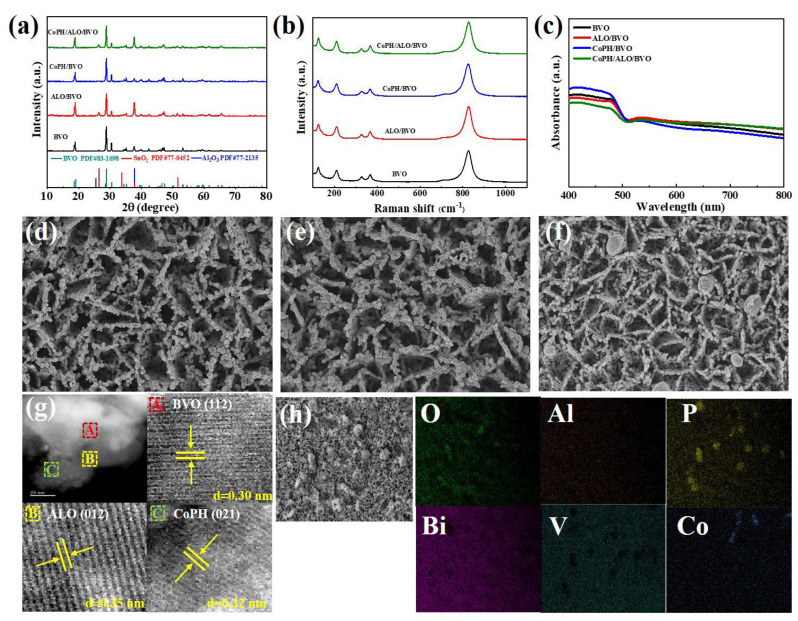
(**a**) XRD patterns, (**b**) Raman spectra, and (**c**) UV-Vis diffused reflectance spectra of BVO, ALO/BVO, CoPH/BVO, and CoPH/ALO/BVO photoanodes. SEM images of (**d**) BVO, (**e**) ALO/BVO, and (**f**) CoPH/ALO/BVO photoanodes. (**g**) HRTEM images and (**h**) EDX mapping of CoPH/ALO/BVO photoanode.

The crystal structures of as-prepared BVO, ALO/BVO, CoPH/BVO, and CoPH/ALO/BVO photoanodes were determined by X-ray diffraction (XRD) in Figure 2a [32]. The diffraction peaks at 18.9°, 28.9°, 30.5°, 35.2°, 40.0°, 47.2°, 50.3°, 53.4°, and 59.4° have been assigned to the (011), (112), (004), (020), (−121), (024), (301), (220), and (132) planes, indicating successful preparation of standard monoclinic BVO (PDF#83-1700). The diffraction peaks at 26.4°, 34.7°, 37.7°, 51.8°, and 65.9° have been assigned to the (110), (101), (200), (211), and (301) planes, which correspond to standard monoclinic SnO_2_ (PDF#77-0452). Those analyses indicate that the BVO had successful growth on FTO substrate. In addition, the diffraction peaks at 37.8° correspond to the (110) facet of standard ALO (PDF#77-2135), suggesting the deposition of the ALO layer on the BVO. Furthermore, no other diffraction peaks were detected, suggesting the low amount of CoPH is consistent with previous findings about metal complexes/semiconductor photoanodes [33]. Though the existence of CoPH is difficult to distinguish, the optical images of these photoanodes (as shown in Figure S1) exhibit the color change (bright yellow for BVO, dark yellow for ALO/BVO, bright yellow for CoPH/BVO, and brown for CoPH/ALO/BVO), indicating the successful loading of ALO and CoPH. To evaluate the phase and presence of CoPH, the CoPH powder was analyzed by an XRD test (Figure S2). The diffraction peaks at 26.0°, 26.9°, 27.4°, 29.4°, 33.8°, 36.1°, 38.5°, 42.6°, 53.6°, 55.3°, 57.8°, 61.9°, and 71.1° have been matched well with the (−112), (−202), (021), (−212), (−221), (−222), (−131), (−313), (−224), (−404), (400), (−503), and (−442) planes of standard monoclinic Co_3_(HPO_4_)_2_(OH)_2_ (PDF#80-1997). It suggests the low content of CoPH in CoPH/BVO and CoPH/ALO/BVO. To further ascertain the structure characteristics of the BVO, ALO/BVO, CoPH/BVO, and CoPH/ALO/BVO photoanodes, Raman spectra were examined within the range of 100–1100 cm^−1^ (Figure 2b). For the BVO, the peak at 826 cm^−1^ belongs to the typical V-O bond, while the peaks at 367 cm^−1^ and 326 cm^−1^ are attributed to the distinct VO_4_^3−^. Moreover, the peaks detected at 124 and 211 cm^−1^ correspond to the external mode of the BVO [34]. Notably, the Raman spectra of the ALO/BVO, CoPH/BVO, and CoPH/ALO/BVO photoanodes are identical to the pristine BVO photoanode, which may be attributed to the low content of ALO and CoPH. The optical properties of these photoanodes were elucidated by UV-Vis diffused reflectance spectra. As shown in Figure 2c, the ALO/BVO, CoPH/BVO, and CoPH/ALO/BVO photoanodes exhibit higher absorption intensity in contrast to the pristine BVO. In addition, all photoanodes possess a similar absorption edge at 506 nm, indicating the negligible effect with the incorporation of ALO and CoPH on light absorption capacity, which is consistent with previous study [35]. Moreover, the UV-Vis spectrum of CoPH displays absorption edges located at 472, 490, 532, 574, and 682 nm, as shown in Figure S3. Both absorption bands can be attributed to the d-d transition of high-spin Co^2+^ in the twisted octahedron [36]. The morphologies of the BVO, ALO/BVO, CoPH/BVO, and CoPH/ALO/BVO photoanodes were investigated by SEM and HRTEM. As illustrated in Figure 2d, the BVO photoanode exhibits a cross-linked worm-like structure. As shown in Figure 2e, negligible morphology change can be observed for the ALO/BVO photoanode, revealing the low content of ALO. For the CoPH/ALO/BVO photoanode, it can be observed that circular block-like CoPH particles are embedded into the photoanode in Figure 2f. To further explore the morphological changes in CoPH with varying concentration, SEM images of CoPH/BVO photoanodes are presented in Figure S4. These photoanodes were prepared using different concentrations of CoPH (0.1 M, 0.2 M, 0.4 M). It can be obtained that the size of CoPH increases with incremental CoPH concentrations, ranging from 0.1 M to 0.4 M. HRTEM was employed to explore the detailed information of the CoPH/ALO/BVO photoanode as presented in Figure 2g. The lattice spacings of 0.30, 0.35, and 0.32 nm correspond to the BVO (112), ALO (012), and CoPH (021) facets, respectively. EDX mapping images (Figure 2h) of the CoPH/ALO/BVO photoanode reveal the existence of O, Al, P, Bi, V, and Co elements, which is consistent with EDX analysis (Figure S5). The elemental compositions were listed in Table S1. These results suggest the uniform distribution of O, Al, P, Bi, V, and Co elements, indicating the successful preparation of the CoPH/ALO/BVO photoanode.

**Figure 3:**
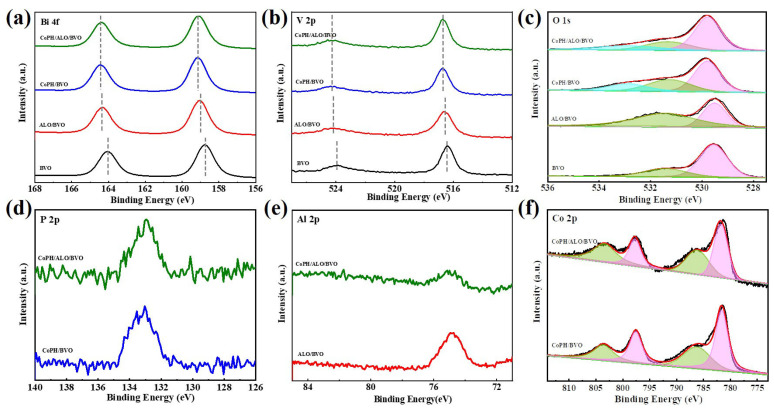
XPS spectra of BVO, ALO/BVO, CoPH/BVO, and CoPH/ALO/BVO photoanodes. (**a**) Bi 4f, (**b**) V 2p, (**c**) O 1s, (**d**) P 2p, (**e**) Al 2p, and (**f**) Co 2p.

X-ray photoelectron spectroscopy (XPS) measurement was carried out to determine the chemical states and surface compositions of BVO, ALO/BVO, CoPH/BVO, and CoPH/ALO/BVO photoanodes. As depicted in Figure 3a, the Bi 4f spectra exhibit distinct peaks at 159.0 and 164.3 eV correspond to Bi^3+^ 4f_7/2_ and Bi^3+^ 4f_5/2_, suggesting the presence of Bi^3+^ cation. In Figure 3b, the peaks at 516.5 and 524.3 eV can be attributed to V 2p_3/2_ and V 2p_1/2_ of V^5+^ species. Compared to the BVO photoanode, the ALO/BVO and CoPH/BVO photoanodes demonstrate that Bi 4f and V 2p peaks shift to higher binding energies (159.0 and 164.3 eV assigned to Bi^3+^ 4f_7/2_ and Bi^3+^ 4f_5/2_, and 516.6 and 524.1 eV attributed to V^5+^ 2p_3/2_ and V^5+^ 2p_1/2_ for ALO/BVO; 159.2 and 164.4 eV assigned to Bi^3+^ 4f_7/2_ and Bi^3+^ 4f_5/2_, and 516.7 and 524.3 eV attributed to V^5+^ 2p_3/2_ and V^5+^ 2p_1/2_ for CoPH/BVO). This finding suggests that there is a mutually attractive chemical interaction facilitating the photoinduced electron transfer between BVO and CoPH/ALO [32,37], thus enhancing PEC performance. This finding can be confirmed by the following EIS test and the LSV measurement. As shown in Figure 3c, the two divided O 1s peaks at 529.6 and 531.4 eV can be assigned to the lattice oxygen of Bi-O bond (purple color) and -OH groups (green color), respectively [38,39]. For the CoPH/BVO and CoPH/ALO/BVO photoanodes, O 1s peak at 532.9 eV assigned to P = O (cyan color) in HPO_4_^2−^ appears, suggesting the successful incorporation of CoPH.

Furthermore, the Al 2p XPS spectra of the ALO/BVO and CoPH/ALO/BVO photoanodes are displayed in Figure 3d. The peak located at 74.7 eV and assigned to Al^3+^ ions in ALO indicates the successful loading of ALO [40]. In Figure 3e, the P 2p XPS spectra of the CoPH/BVO and CoPH/ALO/BVO photoanodes possess two obvious peaks at 133.1 and 134.1 eV, assigned to P 2p_3/2_ and P 2p_1/2_, respectively. This analysis confirms the existence of (HPO_4_)^2−^ in CoPH/BVO and CoPH/ALO/BVO photoanodes, which is likely to enhance the surface reaction kinetics [26]. The Co 2p XPS spectra of the CoPH/BVO and CoPH/ALO/BVO photoanodes are illustrated in Figure 3f. The peaks at 781.6 and 797.6 eV correspond to Co 2p_3/2_ and Co 2p_1/2_ (purple color) of Co^2+^. In addition, two oscillatory satellite peaks (labeled “Sat”, green color) can be observed at 803.5 and 786.4 eV, suggesting the presence of the divalent oxidation state of cobalt in CoPH/BVO and CoPH/ALO/BVO photoanodes [26,41]. This result is consistent with the XRD measurement and confirms the successful incorporation of CoPH in CoPH/BVO and CoPH/ALO/BVO photoanodes [42].

**Figure 4:**
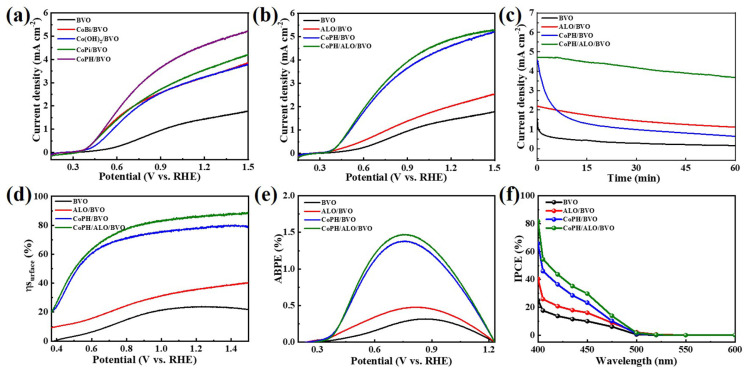
(**a**,**b**) LSV curves of different photoanodes. (**c**) Long-time stability test, (**d**) η_surface_, (**e**) ABPE, and (**f**) IPCE curves of BVO, ALO/BVO, CoPH/BVO, and CoPH/ALO/BVO photoanodes.

Initially, various Co-based OECs including CoBi, CoPi, Co(OH)_2_, and CoPH are employed to a modified BVO photoanode according to previous reports [43,44,45]. Figure 4a demonstrates that the CoPH/BVO photoanode achieves the optimal photocurrent density of 4.7 mA cm^−2^ at 1.23 V_RHE_, which is higher than that of the BVO (1.5 mA cm^−2^), CoPi/BVO (3.6 mA cm^−2^), CoBi/BVO (3.3 mA cm^−2^), and Co(OH)_2_/BVO (3.3 mA cm^−2^) photoanodes. Based on this analysis, the CoPH/BVO photoanode was further optimized with various CoPH concentrations (0.1, 0.2, and 0.4 M) as shown in Figure S6. The maximum photocurrent density was achieved with CoPH concentration at 0.2 M. Then, the as-prepared CoPH/BVO photoanode was selected for further investigation. To further assess the PEC performance of pristine BVO, ALO/BVO, CoPH/BVO, and CoPH/ALO/BVO photoanodes, LSV measurement was performed under illumination (AM 1.5 G, 100 mW cm^−2^). As shown in Figure 4b, the photocurrent densities for BVO, ALO/BVO, CoPH/BVO, and CoPH/ALO/BVO at 1.23 V_RHE_ are 1.5, 2.1, 4.7, and 4.9 mA cm^−2^, respectively. This result indicates the introduction of CoPH and CoPH/ALO can greatly enhance the PEC performance, which may ascribe to the accelerated water oxidation kinetics by CoPH. Table S2 shows the comparisons of PEC performance between previous BiVO_4_-based photoanodes and the CoPH/ALO/BVO photoanode. It can be obtained that CoPH/ALO/BVO exhibits an outstanding PEC performance among these photoanodes. In addition, the stability of the photoanode as an important parameter was evaluated with J-t plots at 1.23 V_RHE_. As illustrated in Figure 4c, it can be obtained that a rapid decay occurs for the BVO photoanode owing to the severe photocorrosion. ALO/BVO and CoPH/ALO/BVO photoanodes exhibit a great improvement on the durable stability (60 min) compared to the BVO photoanode, which may ascribe to the passivation effect induced by the ALO layer.

Furthermore, the surface charge separation efficiencies (*η*_surface_) for these photoanodes were measured with Na_2_SO_3_ as the hole scavenger. As exhibited in Figure 4d, the CoPH/ALO/BVO photoanode demonstrates a charge separation efficiency of 85.9%, which is much higher than that of BVO (23.8%), CoPH/BVO (78.4%), and ALO/BVO (45.9%) photoanodes. This result suggests the modification of CoPH/ALO can greatly promote surface charge separation. Moreover, the applied bias photon-to-current efficiencies (ABPEs) of BVO, ALO/BVO, CoPH/BVO, and CoPH/ALO/BVO photoanodes were calculated and presented in Figure 4e. The CoPH/ALO/BVO photoanode could achieve an ABPE of 1.47% at 0.76 V_RHE_, which is 4.6, 3.1, and 1.1 times that of BVO (0.32%, 0.86 V_RHE_), ALO/BVO (0.48%, 0.81 V_RHE_), and CoPH/BVO (1.38%, 0.75 V_RHE_) photoanodes, respectively. This finding confirms that the modification of CoPH/ALO on the BVO photoanode surface promotes the transfer of photogenerated holes to the photoanode/electrolyte and improves the energy conversion, which improves the PEC performance. This result is also consistent with the LSV measurement and long-time stability test. In addition, the incident photon-to-current conversion efficiency (IPCE) of these photoanodes was calculated as shown in Figure 4f. The IPCE of a CoPH/ALO/BVO photoanode can be up to 81.7% at 400 nm, which is much higher than 22.9%, 45.5%, and 65.7% for BVO, ALO/BVO, and CoPH/BVO photoanodes. These measurements express that the introduction of the CoPH cocatalyst and the ALO passivation layer can improve the surface charge transfer efficiency of BVO, resulting in superior photocurrent density, and the result that is also confirmed by LSV measurement.

**Figure 5:**
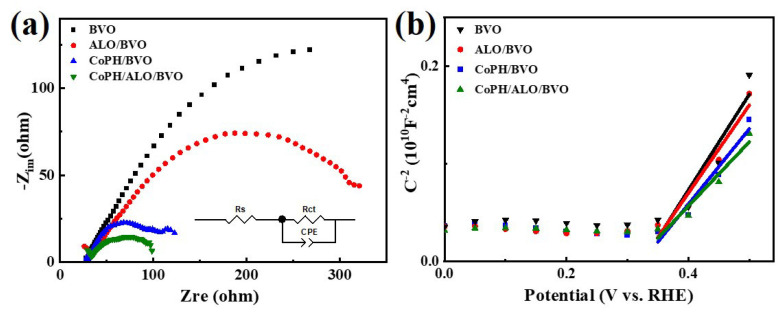
(**a**) EIS Nyquist plots, and (**b**) M-S plots of BVO, ALO/BVO, CoPH/BVO, and CoPH/ALO/BVO photoanodes.

In order to investigate the surface water oxidation kinetics and interfacial charge transfer of BVO, ALO/BVO, CoPH/BVO, and CoPH/ALO/BVO photoanodes, electrochemical impedance spectroscopy (EIS) measurement was performed [44]. The EIS measurements provided us with Nyquist plots for the BVO, ALO/BVO, CoPH/BVO, and CoPH/ALO/BVO photoanodes, as depicted in Figure 5a. These plots were analyzed using an equivalent mode. In this model, *R_s_* and *R_ct_* represent the solution resistance and charge transfer resistance of the electrolyte, respectively. According to the fitted values of the BVO, ALO/BVO, CoPH/BVO, and CoPH/ALO/BVO photoanodes, CoPH/ALO/BVO demonstrates a lower *R_ct_* (92.0 Ω) than those of BVO (566.7 Ω), ALO/BVO (364.2 Ω), and CoPH/BVO (98.5 Ω), indicating that the introduced CoPH/ALO could facilitate the interfacial charge transfer and accelerate the water oxidation kinetics. This analysis is consistent with the order of LSV measurement. Our findings, as demonstrated by these EIS results, provide a deeper understanding of the mechanisms at play in these prepared photoanodes. This result is vital for the future design and optimization of photoanodes for PEC applications, paving the way for more efficient and sustainable solar fuel generation technologies. The Mott–Schottky (M-S) plots of the BVO, ALO/BVO, CoPH/BVO, and CoPH/ALO/BVO photoanodes are displayed in Figure 5b. All photoanodes exhibit positive slopes, indicating the n-type feature of the BVO semiconductor [46]. The charge carrier densities for the BVO, ALO/BVO, CoPH/BVO, and CoPH/ALO/BVO photoanodes were estimated to be 2.1 × 10^18^, 2.3 × 10^18^, 2.7 × 10^18^, and 3.2 × 10^18^ cm^−3^, respectively. The increased carrier density of CoPH/ALO/BVO can be attributed to the facilitated interfacial charge transfer by the introduction of CoPH/ALO, which is consistent with the EIS results. Additionally, the findings also confirm the positive effect that the introduction of the CoPH cocatalyst and the ALO passivation layer has on the BVO photoelectrode, significantly improving the PEC performance. The charge carrier densities of the BVO, ALO/BVO, CoPH/BVO, and CoPH/ALO/BVO photoanodes were estimated to be 2.1 × 10^18^, 2.3 × 10^18^, 2.7 × 10^18^, and 3.2 × 10^18^ cm^−3^, respectively. It can be observed that CoPH could obviously increase the carrier density. Notably, the N_d_ of the ALO/CoPH/BVO photoanode is 1.5 times that of the pristine BVO, indicating the enhanced electronic conductivity with the introduction of the ALO passivation layer and CoPH OEC. This enhancement could result in the accelerated electron–hole separation and promoted electron transport property.

**Figure 6:**
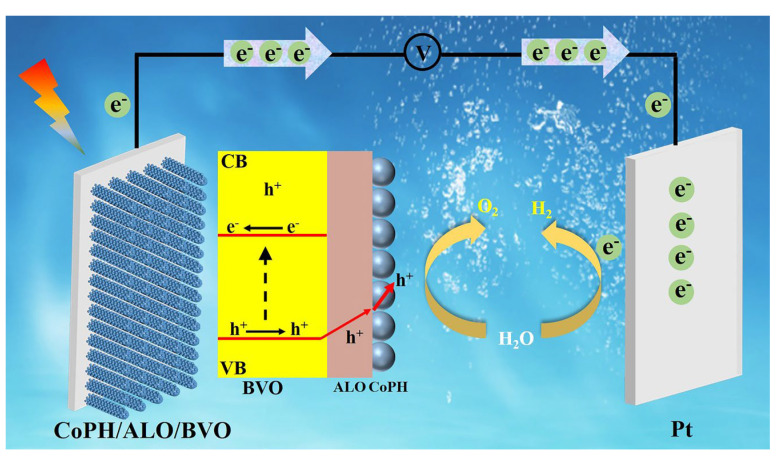
A proposed mechanism of the CoPH/ALO/BVO photoanode.

Based on the above PEC analyses, we have proposed a mechanism for PEC water splitting as illustrated in Figure 6. Under light illumination, the electron–hole pairs are generated and separated at the BVO layer. The holes are transferred to the surface of the photoanode for the water oxidation, and the electrons are flowed to the Pt electrode for the hydrogen evolution. The observed PEC enhancement can be primarily ascribed to several critical factors. First of all, CoPH combines the advantages of cobalt oxide and cobalt phosphide to increase the conductivity, thus improving the photocurrent density [26]. This enhancement can be confirmed by LSV tests. Secondly, EIS and M-S measurements reveal the decreased transfer resistance and the increased carrier density with CoPH modification. This suggests that the CoPH incorporation accelerates the water oxidation kinetics and facilitates the interfacial charge transfer, further improving the PEC performance. However, CoPH/BVO still suffers from severe photocorrosion, leading to a rapid decay in photocurrent density. With the introduction of an ALO passivation layer, the stability can be obviously improved. It can ascribe to the reduced photocorrosion with the passivated surface defect state [28]. The presence of a negative charge on the ALO promotes the transfer of photogenerated holes, thereby inhibiting the surface charge recombination [47]. This improvement is evident from the η_surface_ and EIS tests. In conclusion, the BVO photoanode achieves optimal PEC performance under the synergistic effect of the CoPH cocatalyst and the ALO passivation layer.

## 3. Experimental Section

### 3.1. Preparation of Photoanodes

#### 3.1.1. Synthesis of BVO Photoanode

In a standard procedure, 3.32 g of KI were dissolved in 50 mL H_2_O, and the PH was adjusted to 1.7 using HNO_3_ while stirring in an ice bath. Subsequently, 970 mg of Bi(NO_3_)_3_·5H_2_O was added to the solution, and it was stirred for 1 h. Afterward, the solution was then mixed with 20 mL of absolute ethanol containing 0.23 M p-Benzoquinone and stirred for 1 h to create an electrolyte. For the electrodeposition, a typical three-electrode cell was utilized. The working electrode was conducted from FTO (1 cm × 2 cm), the reference electrode was Ag/AgCl (4 M KCl), and the counter electrode was Pt foil (1 cm × 1 cm). The deposition area on the FTO was confined to 1 cm × 1 cm. Electrodeposition was performed at −0.1 V vs. Ag/AgCl to produce BiOI, with a charge of 0.3 C cm^−2^ passing through the system. Subsequently, a 75 μL DMSO solution containing 0.2 M VO(acac)_2_ was applied to the BiOI surface, followed by calcinating at 450 °C for 2 h to convert BiOI to BVO. Following cooling to room temperature, the BVO material was immersed in a 1 M NaOH solution for 20 min under gentle agitation, then rinsed with DI water and dried.

#### 3.1.2. Synthesis of ALO/BVO Photoanode

Al_2_O_3_ layer was synthesized by immersion–calcination method. The BVO photoanode was immersed in a 10 mM AlCl_3_ aqueous solution for 2 min and subsequently calcinated in air at 200 °C for 0.5 h.

#### 3.1.3. Synthesis of CoPH/BVO Photoanode

CoPH/BVO was synthesized by hydrothermal method. 3 mM Co(NO_3_)_2_·6H_2_O and 2 mM K_2_HPO_4_ were mixed in 15 mL distilled water and stirred for 15 min. The reaction mixture and BVO photoanode were transferred to a 25 mL Teflon-lined hydrothermal vessel and then placed in an oven at 180 °C for 12 h. After the completion of the reaction, it was washed twice with distilled water and subsequently dried in an oven at 60 °C for 20 min.

#### 3.1.4. Synthesis of CoPH/ALO/BVO Photoanode

The CoPH/ALO/BVO photoanode was prepared according to the above steps (synthesis of CoPH/BVO photoanode), except for changing BVO to ALO/BVO into Teflon-lined hydrothermal vessel.

#### 3.1.5. Synthesis of CoBi/BVO Photoanode

First, CoBi/BVO photoanode was synthesized by a facile electrodeposition method in a standard three-electrode cell with 1 M potassium borate electrolyte and 0.5 mM Co(NO_3_)_2_·6H_2_O. Platinum wire and Ag/AgCl electrodes were employed as the counter and reference electrodes, respectively. Subsequently, under AM 1.5 G illumination, CoBi was electrodeposited on BVO photoanode at 1.2 V_RHE_ for 5 s.

#### 3.1.6. Synthesis of Co(OH)_2_/BVO Photoanode

First, BVO photoanode was immersed in a 5 mM CoCl_2_ 6H_2_O solution for 10 h. After that, it was removed and dried in an oven at 60 °C for 30 min, and marked as Co(OH)_2_/BVO.

#### 3.1.7. Synthesis of CoPi/BVO Photoanode

CoPi/BVO photoanode was synthesized with electrodeposition method. The electrolytes were prepared by dissolving 0.5 mM Co(NO_3_)_2_·6H_2_O in 0.1 M potassium phosphate (KPi) buffer solution. Electrodeposition was carried out at 1.5 V_RHE_ for 30 s. After that, it was removed and dried in an oven at 60 °C for 30 min, and marked as CoPi/BVO.

### 3.2. Characterization

The crystallinity and structure were carried out by X-ray diffraction analysis (XRD, Rigaku Smart Lab, Tokyo, Japan). The elemental composition and chemical state of the materials were explored by X-ray photoelectron spectroscopy (XPS, Thermo Fisher Scientific K-Alpha+, Waltham, MA, USA). The C 1 s peak with a binding energy of 284.8 eV was used to correct the binding energies of other elements. Surface morphology and elemental distribution were further investigated by scanning electron microscopy (SEM, Zeiss Sigma300, Jena, Germany) equipped with an energy dispersive X-ray spectrometer (EDS). The samples were evaporated with Pt for 60 s before SEM measurements. Optical properties were analyzed by ultraviolet–visible spectroscopy (UV-Vis, PerkinElmer lambda 950, Hongkong, China).

### 3.3. Photoelectrochemical Measurement

All photoelectrochemical measurements were tested in a standard three-electrode cell with an electrochemical workstation (CHI760E, Shanghai Chenhua Instrument Co., Ltd., Shanghai, China). The photoanode was used as working electrode, Ag/AgCl (4 M KCl) was used as reference electrode, Pt foil (1 cm × 1 cm) was used as counter electrode, and 1 M KBi buffer (pH = 9) was used as electrolyte. A 300 W xenon lamp (PLS-SEX300, Beijing Perfect Light Technology Co., Ltd., Beijing, China) equipped with an AM 1.5 G filter was used as the light source, and the incident light intensity was calibrated to 100 mW/cm^2^. Linear sweep voltammogram (LSV) was measured at a scan rate of 10 mV s^−1^. The frequency range for photoelectrochemical impedance spectroscopy (PEIS) was 100 kHz to 0.1 Hz. The Nyquist plot was fitted to the corresponding equivalent circuit by Z-view 2 software.

The carrier density (N_d_) is calculated using the following equation:

N_d_ = (2/e_0_εε_0_) [d(1/C^2^)/dV]^−1^

In the given equation, C indicates the capacitance of the space charge region, e_0_ indicates the electron charge (1.602 × 10^−19^ C), ε indicates the permittivity of BVO (68), ε_0_ is the vacuum permittivity (8.854 × 10^−14^ F/cm), and V is the potential applied to the electrode.

The applied bias photon current efficiency (ABPE) is computed using the equation provided:

ABPE = ((1.23 − V_b_) × J/P_light_) × 100%

where V_b_ denotes the applied potential versus RHE, while J is the observed photocurrent density (mA cm^−2^). P_light_ refers to the optical density (100 mW cm^−2^).

The incident photon-to-current conversion efficiency (IPCE) is calculated according to the formula:

IPCE = (1240 × J)/(λ × P_light_) × 100%

In the formula, J represents the photocurrent density (mA cm^−2^) measured at a certain wavelength, λ is the wavelength of the incident light (nm), and P_light_ refers to the intensity of the power at a specific wavelength (mW cm^−2^).

The surface charge separation efficiency (η_surface_) is calculated based on the following formula:

η_surface_ = J_H2O_/J_Na2SO3_

where J_H2O_ refers to the photocurrent density of PEC water oxidation, while J_Na2SO3_ is the photocurrent density achieved when using Na_2_SO_3_ as the sacrificial agent.

## 4. Conclusions

In summary, we have successfully prepared a CoPH/ALO/BVO photoanode, demonstrating a significant enhancement in PEC performance. The introduction of the CoPH cocatalyst and the ALO passivation layer onto the BVO photoanode has resulted in a remarkable improvement in efficiency and stability in PEC water splitting. The CoPH/ALO/BVO photoanode achieves an impressive photocurrent density of 4.9 mA cm^−2^ at 1.23 V_RHE_, representing a profound 3.3-fold enhancement over the pristine BVO. This notable enhancement in PEC performance is primarily due to the combination of the passivation effect induced by the catalytical activity provided by CoPH and the ALO layer. This work offers a new idea to design the high-quality photoanode with the synergetic strategy combining the OEC and passivation effect.

## Data Availability

Data are contained within the article and Supplementary Materials.

## Supplementary Materials

The following supporting information can be downloaded at: https://www.mdpi.com/article/10.3390/molecules29030683/s1, Figure S1: Optical images of these photoanodes; Figure S2: XRD patterns of CoPH; Figure S3: UV-Vis spectrum of CoPH; Figure S4: SEM images of CoPH/BVO photoanodes with different concentrations of CoPH; Figure S5: EDX pattern of CoPH/ALO/BVO photoanode; Table S1: Elemental composition of ALO/CoPH/BVO; Figure S6: LSV curves of BVO photoanodes with different concentrations of CoPH; Table S2: The PEC performance comparison between previous reports and this work.
