# Supplementary material for: Synergistic Effect of Co3(HPO4)2(OH)2 Cocatalyst and Al2O3 Passivation Layer on BiVO4 Photoanode for Enhanced Photoelectrochemical Water Oxidation
Source: Molecules. 2024 Feb 1;29(3):683. doi: 10.3390/molecules29030683 (PMC10856029; doi:10.3390/molecules29030683)
Supplement: Supplementary file 1 [file molecules-29-00683-s001.zip › molecules-2823587-supplementary.pdf]

Supporting information

Synergistic Effect of $Co_3(HPO_4)_2(OH)_2$ Cocatalyst and Al_2O_3 Passivation Layer on $BiVO_4$ Photoanode for Enhanced Photoelectrochemical Water Oxidation

Zijun Sun, Zhen Li, Jinlin Chen, Yuying Yang, Chunrong Su, Yumin Lv, Zhenhong Lu, Xiong He and Yongqing Wang *

Guangxi Key Laboratory of Multidimensional Information Fusion for Intelligent Vehicles, School of Electronic Engineering, Guangxi University of Science and Technology, Liuzhou 545000, China

* Correspondence:wangyongqing1991@163.com

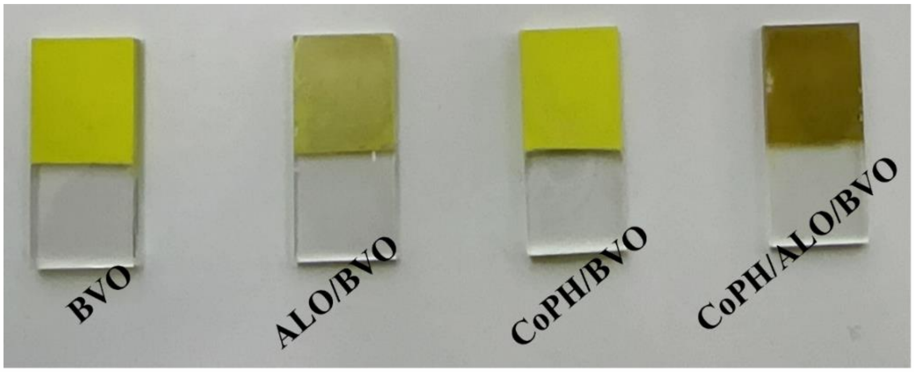

Figure S1 Optical images of these photoanodes.

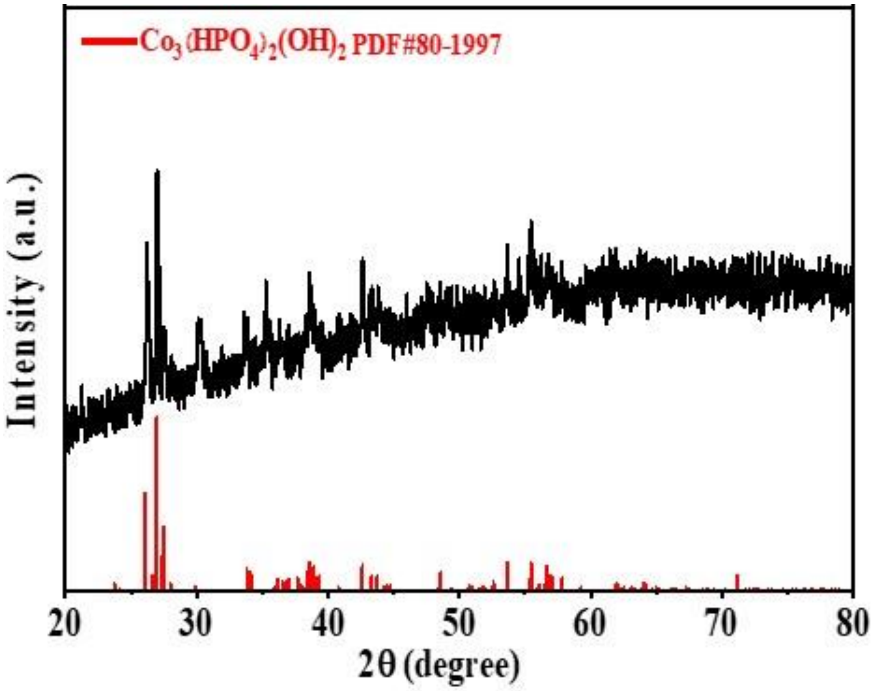

Figure S2 XRD patterns of CoPH.

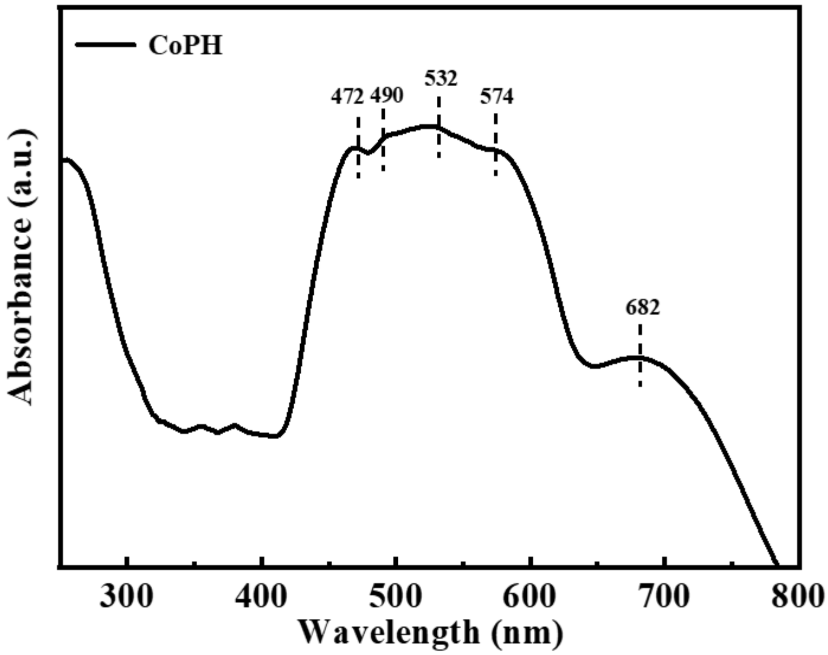

Figure S3 UV-Vis spectrum of CoPH.

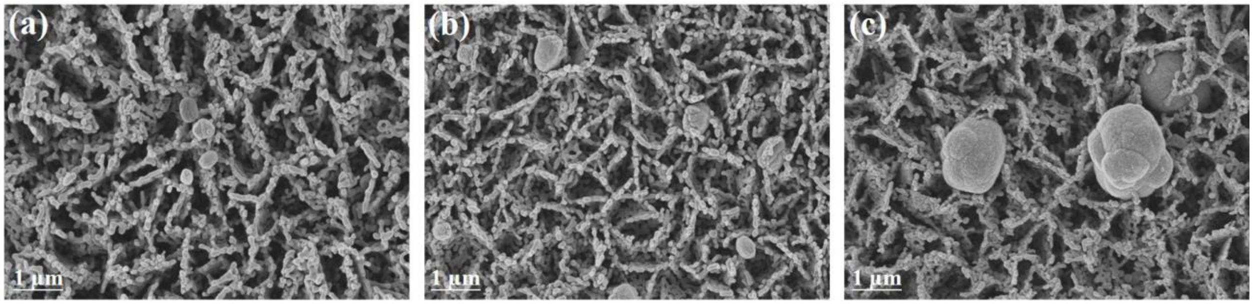

Figure S4 SEM images of CoPH/BVO photoanodes with different concentrations of CoPH.

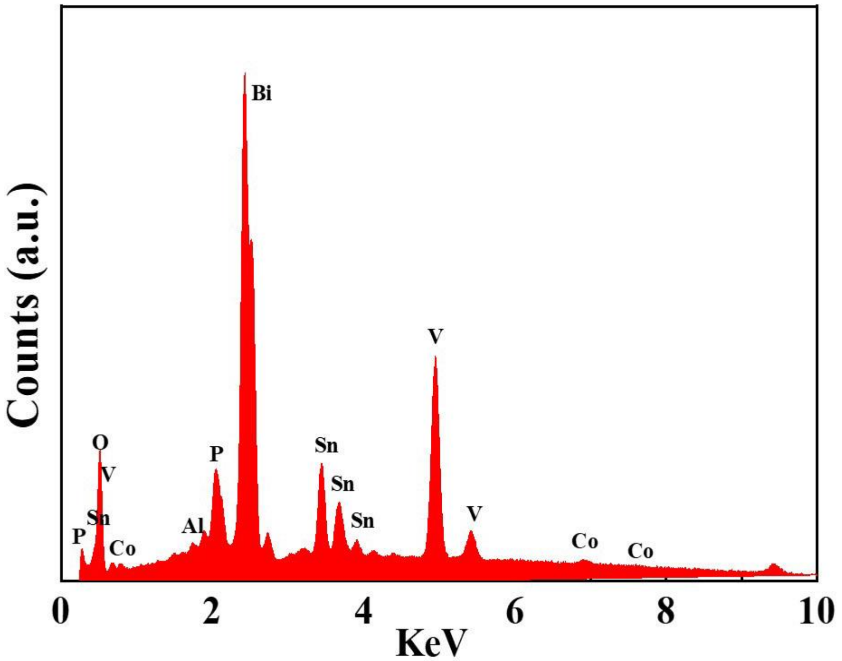

Figure S5 EDX pattern of CoPH/ALO/BVO photoanode.

Table S1. Elemental composition of ALO/CoPH/BVO

**Element**	**Weight %**	**Atomic %**
O K	11.83	45.66
Al K	1.00	2.29
P K	2.88	5.75
Bi M	42.23	12.48
Sn L	24.60	12.81
V K	16.41	19.90
Co K	1.05	1.10

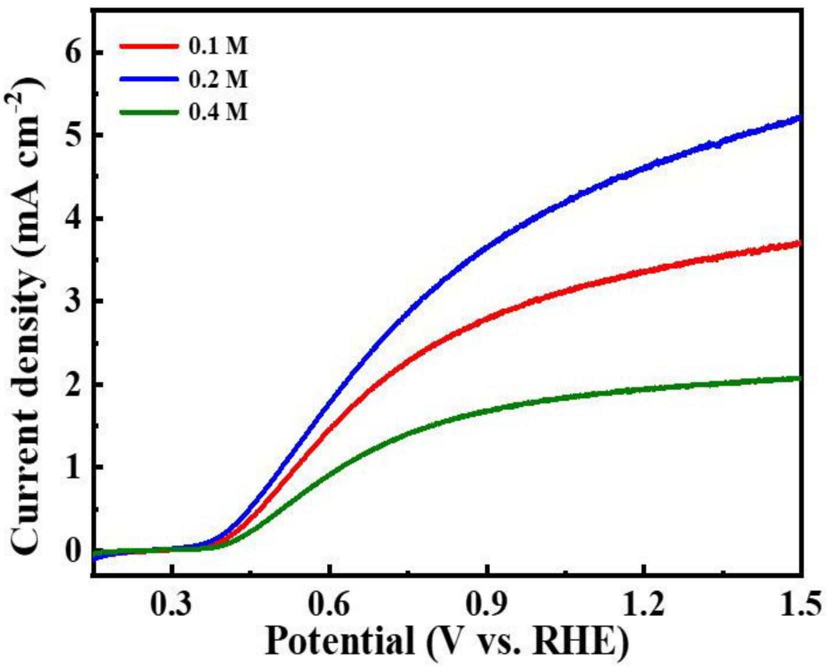

Figure S6 LSV curves of BVO photoanodes with different concentrations of CoPH.

Table S2 The PEC performance comparison between previous reports and this work.

Photoanode	Photocurrent density (at 1.23 V vs. RHE)	Ref.
**CoPH/ALO/BVO**	**4.9 mA cm^{-2}**	**This work**
α-Fe_2O_3/Al_2O_3/$CuCoO_x$	2.2 mA cm^{-2}	Applied Catalysis B: Environmental, 2020, 277, 119197.
$CoPc(NH_2)_4$/$BiVO_4$	3.0 mA cm^{-2}	Applied Surface Science, 2021, 564, 150463.
NiFe-LDH/MoO_x/$BiVO_4$	2.7 mA cm^{-2}	Journal of Colloid and Interface Science, 2022, 626, 146-155.
$BiVO_4$-Ni/Co_3O_4	2.2 mA cm^{-2}	Applied Surface Science, 2021, 538, 148150.
$BiVO_4$/CoAl-LDH	3.5 mA cm^{-2}	Applied Catalysis B-environmental, 2021, 286, 119875.
$BiVO_4$/g-C_3N_4/CoOOH	4.2 mA cm^{-2}	Journal of Power Sources, 2021, 494, 229701.
$BiVO_4$/Vo-FeNiOOH	3.8 mA cm^{-2}	Small, 2022, 18, 2107938.
FeOOH/Ni-$BiVO_4$	2.5 mA cm^{-2}	Journal of Colloid and Interface science, 2023, 640, 162-169.
$BiVO_4$/CoP	4.1 mA cm^{-2}	International Journal of Hydrogen Energy, 2021, 46, 15517-15525.
Mn-FeOOH/$BiVO_4$	1.4 mA cm^{-2}	Journal of Alloys and Compounds, 2022, 894, 162571.
